# Notes and News

**Published:** 1894-10-13

**Authors:** 


### Notes and News.

Collected and Communicated.

The Royal Surrey County Hospital is to have the aid of the Duke of Connaught on October 27th, when he will preside at a meeting at Guildford in aid of the institution.

The following members of the Ladies' Committee of the Chelsea Hospital for Women have tendered their resignations to the board of management, viz.: Mrs. R. P. Ebden (vice-president), the Hon. Flora Macdonald, Mrs. Brend Batten, Miss Dudin Brown, Mrs. Edis, Mrs. Furley, Mrs. Hussey-Walsh, Mrs. Newton, Mrs. Reeves, Mrs. Alexander Ross, Mrs. F. F. Schackh, Mrs. Travers, and Mrs. Webb-Peploe.

During the International Congress of Hygiene at Buda-Pesth a comparison between the Æsculap Bitter Water and all other aperient waters was invited. The Æsculap Spring is owned and supervised by Englishmen, and it is gratifying to learn that its water was found to be the best put up of all the Hungarian natural waters exhibited at Buda-Pesth. The efficacy of the Æsculap water is widely recognised by the profession, and it is important to know that when bottled it is the clearest and purest of all Hungarian natural waters.

We understand that Sir Savile Crossley, Bart., has most generously purchased from the Salvation Army, at a cost of £3,000, and presented to the Eastern Counties' Asylum for Idiots, Colchester, the house known as "Oceanville," Clacton-on-Sea, for use as a seaside home for their patients. It is admirably suited for this purpose, has a commanding view of the sea, and stands in its own grounds of nearly an acre. As it is the house in which Mrs. Booth died the Salvation Army were only induced to part with in consideration of the good object for which it was intended to be used. For some time the need of a seaside home has been greatly felt by the Board of Directors, and it will prove a most valuable acquisition to the resources of the asylum. Mr. A. M. White, the hon. solicitor of the institution, kindly conveyed the property free of charge.

The Duke and Duchess of York opened the Medical School in connection with the Yorkshire College at Leeds on the 5th inst.

In a paper marked by great vividness of description and wealth of illustration Dr. J. Althaus described to the opening meeting of the Medical Society of London the various phases of hypochondriasis. The point on which he laid especial emphasis was the reality of the sufferings of the patient, and yet their subjective nature. On the one hand it had to be distinguished from melancholia and other forms of delusional insanity, on the other from nosophobia. It was a mistake to imagine that because the most careful examination discovered no disease therefore the whole thing was a delusion. However false the patient's deduction as to the nature of the disease might be, the sensations which he so tried to explain were real enough, and probably depended on some error, either of nutrition or development, in some portion of the still unalloted area in the brain cortex. In this lay the great difference, both as to treatment and prognosis, between hypochondriasis and nosophobia. In the latter, however great might be dread of some disease, it sprung from some extrinsic cause, some morbid condition in the part complained of, or some suggestion from without. Hence in cases of nosophobia reassurance, and treatment applied to the seat of the suffering, often at once removed the dread; whereas in hypochondriasis treatment of the part complained of was commonly of but small avail, the malady really being a neurosis having its origin in the brain; while reassurance, based on a disbelief in the reality of the sensation, only confirmed the patient in his idea that his case was not properly understood.

Though the afternoon was of the dampest and murkiest quite a large number of visitors found their way into Surrey on Saturday last to be present at the laying of the foundation-stone, by the Lord Mayor, of the Caxton Convalescent Home, on Limpsfield Chart. The comfort of the guests was, in some respects, cared well for, but it was unfortunate that in such uncertain weather better covert had not been provided at the scene of the actual ceremony, and the carelessness on the part of those responsible which led to a wait of ten minutes on saturated ground while the stone was with difficulty coaxed into position must have justly annoyed the Lord Mayor. The Caxton Home is intended for the benefit of sick and ailing members of the "printing and allied trades." It was originally proposed to erect a portion of the home at Swanage, capable of accommodating 12 patients, but Mr. Passmore Edwards having generously offered to defray the cost of this part of the building, and having suggested the advisability of placing it nearer London, the committee ultimately secured the present site. There will be about 10 acres of ground in all, and the building is eventually to contain 50 beds. The home is to be established upon a self-supporting basis, and subscriptions are being earnestly invited from all members of the various trades who are to profit by it, with, so far, a very fairly satisfactory result. The committee are really to be congratulated upon the position they have chosen for the home. The building (of which Mr. Saxon Snell, F.R.I.B.A., is the architect) will face south-east, protected by the rising Downs at the back from cold winds, and having in front a grand view over the beautiful scenery for which Surrey is famous.

